# Health Notes Audit

**DOI:** 10.1192/bjo.2024.570

**Published:** 2024-08-01

**Authors:** Charlotte Golding, Neeti Sud

**Affiliations:** Cumbria, Northumberland, Tyne and Wear NHS Foundation Trust, Newcastle upon Tyne, United Kingdom

## Abstract

**Aims:**

To assess whether patients admitted to the forensic secure rehabilitation ward are transferred with their physical health notes.

Most patients admitted to secure rehabilitation do not have an open GP record due to last registration with primary care having been many years previous as a result of a lengthy prison/hospital stay. Additionally, patients may be referred from an out of area prison or hospital. A comprehensive psychiatric history paperwork is obtained at referral. This audit was to assess how many patients currently on the rehabilitation ward arrived with complete physical health notes. We defined a complete set of physical health notes to mean:

1. Records from medical consultations linked to physical health during time in prison or psychiatric hospital.
2. Any physical health history prior to current incarceration/admission episode from primary and secondary care.
3. Complete prescription of physical health related medications including allergies, doses, regime, and indication.

**Methods:**

Retrospective review of patient electronic records sent by discharging institution when the patient was transferred to the rehabilitation ward.

Data collected: List of documentation of patient's physical health records around transfer time. Identification of the contents of the records provided by the transferring ward.

We then compared the information available to our criteria for complete physical health notes.

Participants: All current residents of the male secure rehabilitation ward (n = 12) were included.

**Results:**

7 out of the 12 patients included were transferred to the secure ward with notes that fulfilled the criteria as set by audit team.

Two patients were transferred with only the prescription of current medications. There was however, a brief physical health summary in care coordination notes sent earlier.

One patient was transferred with the prescription and a brief list of their past medical history.

The remaining 2 patients were transferred without any formal physical health documentation prior to transfer, however, they were transferred from an adjacent ward and therefore, all records were already on the electronic records. There was no formal verbal or written physical health handover.

**Conclusion:**

It is important for our ward to ensure we have comprehensive and complete physical health summary for each patient on admission.

A proforma will be used at preadmission meetings from February 2024 to request specific information from discharging wards. We will re-audit in February 2025 to assess improvement in records requested and obtained.

